# Attitudes and perceptions of UK medical students towards artificial intelligence and radiology: a multicentre survey

**DOI:** 10.1186/s13244-019-0830-7

**Published:** 2020-02-05

**Authors:** Cherry Sit, Rohit Srinivasan, Ashik Amlani, Keerthini Muthuswamy, Aishah Azam, Leo Monzon, Daniel Stephen Poon

**Affiliations:** 1 Department of Radiology, Guy’s and St. Thomas’ NHS Foundation Trust, London, UK; 2 Department of Interventional Radiology, Guy’s and St. Thomas’ NHS Foundation Trust, London, UK; 3 School of Medicine, Faculty of Medicine and Life Sciences, King’s College London, London, UK

**Keywords:** Artificial intelligence, Education, Medical student, Radiology

## Abstract

**Objectives:**

To explore the attitudes of United Kingdom (UK) medical students regarding artificial intelligence (AI), their understanding, and career intention towards radiology. We also examine the state of education relating to AI amongst this cohort.

**Methods:**

UK medical students were invited to complete an anonymous electronic survey consisting of Likert and dichotomous questions.

**Results:**

Four hundred eighty-four responses were received from 19 UK medical schools. Eighty-eight percent of students believed that AI will play an important role in healthcare, and 49% reported they were less likely to consider a career in radiology due to AI. Eighty-nine percent of students believed that teaching in AI would be beneficial for their careers, and 78% agreed that students should receive training in AI as part of their medical degree. Only 45 students received any teaching on AI; none of the students received such teaching as part of their compulsory curriculum. Statistically, students that did receive teaching in AI were more likely to consider radiology (*p* = 0.01) and rated more positively to the questions relating to the perceived competence in the post-graduation use of AI (*p* = 0.01–0.04); despite this, a large proportion of students in the taught group reported a lack of confidence and understanding required for the critical use of healthcare AI tools.

**Conclusions:**

UK medical students understand the importance of AI and are keen to engage. Medical school training on AI should be expanded and improved. Realistic use cases and limitations of AI must be presented to students so they will not feel discouraged from pursuing radiology.

## Key points

- UK medical students do not feel adequately prepared to work alongside AI, but understand the increasing importance of AI in healthcare and would like to receive teaching on the subject
- A significant number of UK medical students are discounting radiology as a possible career due to AI. Students that received AI teaching were less likely to rule out a career in radiology
- Although the small number of students that received AI teaching felt more confident in working with AI in the future compared to students that did not receive teaching, a significant number of taught students still felt inadequately prepared.

## Introduction

The application of artificial intelligence (AI) in healthcare continues to generate increasing interest. Within radiology, there are now AI tools that employ deep learning methods such as convolutional neural networks (CNNs) which are effective at performing narrow classification tasks where large training datasets are available. High profile examples include the classification of chest radiographs based on abnormality for triage [1] or pathological processes [2, 3]. Multiple studies have demonstrated the potential utility of such AI algorithms in other medical specialties including ophthalmology [4], dermatology [5], and pathology [6]. Although there is currently a paucity of evidence to support routine use of AI algorithms in real-world clinical practice, it is expected that with increasing academic and industry interest, validated use cases for AI tools in radiology will likely emerge rapidly [7].

As the field of healthcare AI continues to expand, it is increasingly apparent that AI education for clinicians and medical students is needed. In the United Kingdom (UK), the use of novel digital methods in health has been the subject of a recent national governmental review [8]. Of note, the Topol review places explicit emphasis on the importance of arming the current and future clinical workforce with the necessary skills to work critically with novel digital tools, including those underpinned by AI. Currently, UK medical schools are not mandated to include this in the curriculum by the General Medical Council. There exists some anecdotal evidence that certain UK medical schools do provide limited educational opportunities in healthcare AI; however, the actual prevalence of such training and the level of AI literacy amongst the UK medical student population remains unknown. Furthermore, to effectively engage medical students on this topic, it would be useful to understand their perception towards AI as a cohort. It is not unreasonable to assume the current environment of enthusiasm towards AI exerts some influence on medical student attitude and behaviour. Of specific relevance to radiology, this has been previously studied by groups in Canada [9] and Germany [10], where multi-centre surveys were conducted on medical students from their respective countries. The Canadian group found that a significant proportion of Canadian medical students were less likely to consider radiology as a career due to fear of replacement. Although the German group reported that only a minority of students believed that AI would replace radiologists, actual career intentions were not examined. We are unaware of any published studies investigating the views and attitudes that UK medical students hold towards AI, nor are we aware of any European study that examines the influence exerted upon medical students by the development of AI.

In this study, we aim to understand UK medical students’ views on AI and explore if AI influences their career intentions with specific regard to radiology. We also collated information on medical students’ understanding of AI and assessed their level of confidence in working alongside AI in the future.

## Materials and methods

Students from UK medical schools were invited to complete an electronic survey (Google Forms, Google LLC) designed jointly by clinicians with an interest in AI and/or medical education. Contact was made with all UK medical schools with a request to circulate the survey to all currently enrolled students. Students were also directly invited to complete the survey via social media. Students were required to enter their institutional email address at the start of the survey, and only individuals with a valid UK medical school email address were able to participate; all responses were subsequently anonymised. It was made explicitly clear to participants at the start of the survey that their responses were anonymous. Minimal risk ethical approval was obtained from the King’s College London research ethics office (KCL-MRA18/19-11127). Informed consent was confirmed with individual participants at the start of the survey.

The survey design underwent several rounds of iteration, and final validation was performed with a group of 30 medical students from King’s College London who were not included in the final survey. The final survey consisted of 11 5-point Likert questions, whereby participants rated their agreement towards a presented statement relating to their current attitudes towards AI, their career intentions towards radiology, their current understanding of AI, and their confidence in using AI tools in a routine and critical manner following graduation. Dichotomous questioning was used to determine if participants received teaching on AI and if this teaching formed a compulsory part of their curriculum. The questionnaire is attached as Additional file 1.

Statistical analysis was performed using R (ver. 3.5.3; Great Truth) [11] with RStudio (ver. 1.2.1335) [12]. Simple descriptive statistics are presented in percentages. An unpaired two-tailed Wilcoxon rank-sum test was performed to compare the responses relating to career choice and perceived competence in post-qualification use of AI tools between the group of students which received teaching in AI versus the group of students that did not. A *p* value of less than 0.05 was considered statistically significant.

## Results

**Fig. 1:**
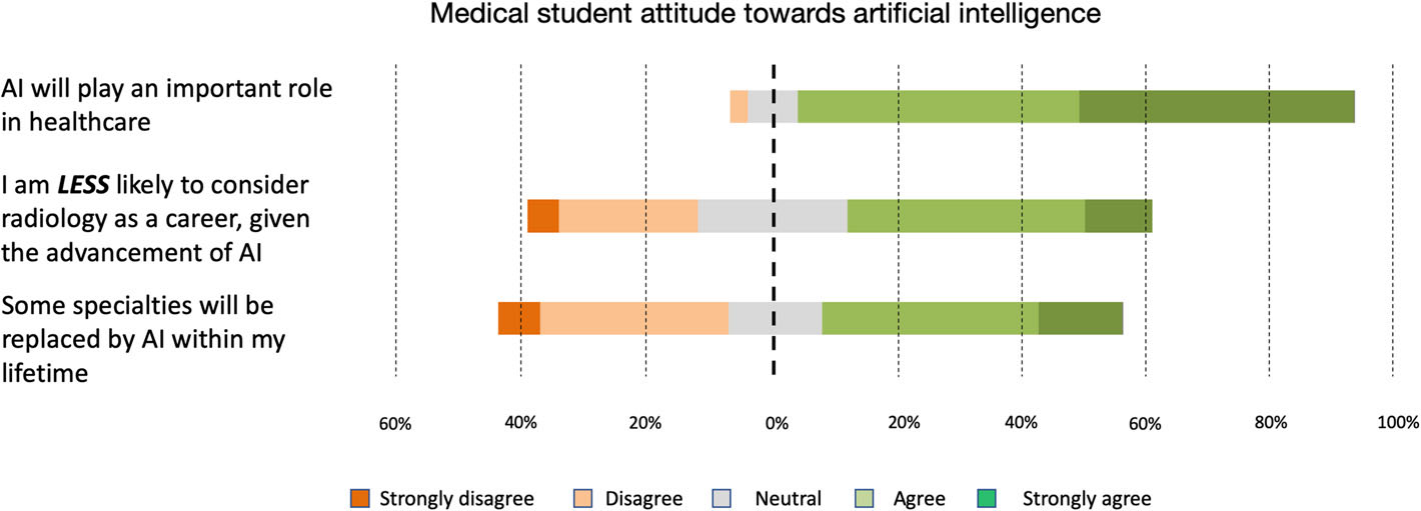
Summary of questions relating to medical student attitude towards AI

A total of 484 responses were received from 19 out of 34 UK medical schools currently awarding medical degrees recognised by the General Medical Council. The majority (88%, *n* = 432) of respondents believe that AI will play an important role in healthcare in the future, rating their responses as either strongly agree (44.2% *n* = 214) or agree (45% *n* = 218). Just under half of the respondents (strongly agree or agree, 49.2% *n* = 238) reported they were less likely to consider a career in radiology due to AI, compared to those who do not feel they are less likely to consider a career in radiology due to AI (strongly disagree or disagree, 27.1% *n* = 131). A similar number of students also believed that some specialities will be replaced by AI within their lifetime (strongly agree/agree, 48.3% *n* = 234; strongly disagree/disagree, 36.8% *n* = 178) (Fig. 1).

**Fig. 2:**
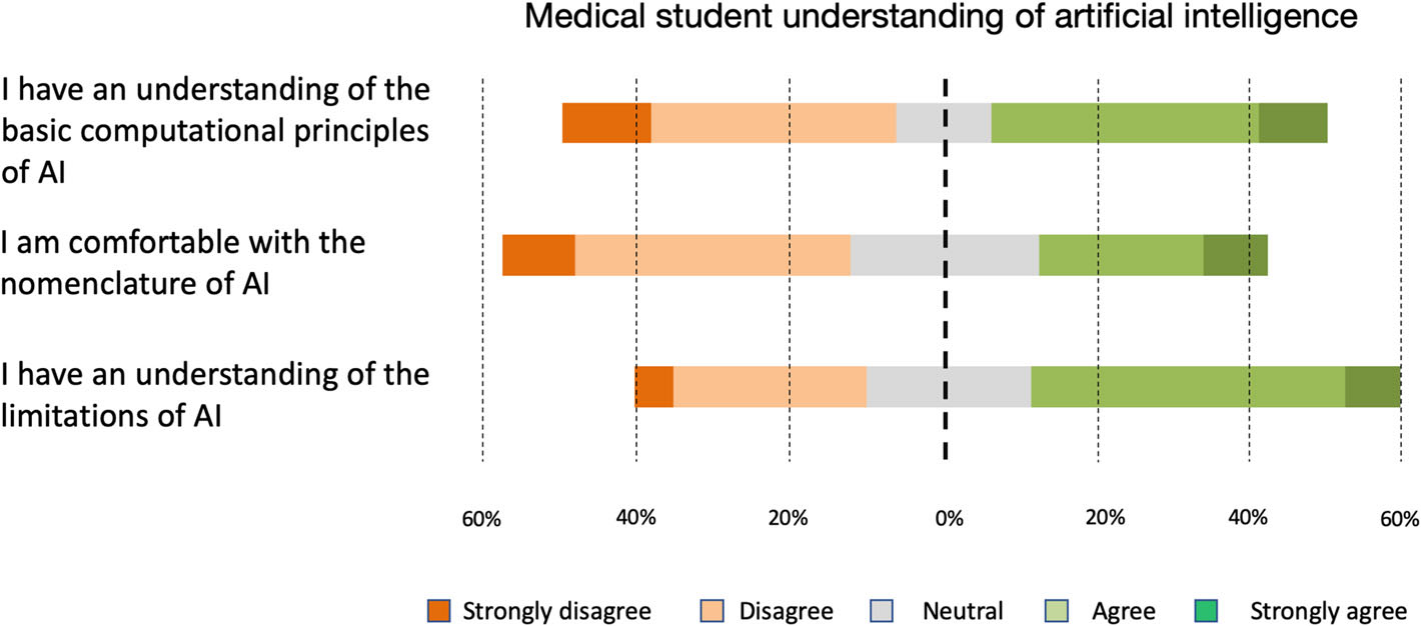
Summary of questions relating to medical student understanding of AI

With regard to the questions relating to the current understanding of AI, nearly half the respondents selected responses indicating they have an understanding of the basic computational principles that underpin AI; 44.6% (*n* = 214) of respondents selecting strongly agree or agree, whilst 43.4% (*n* = 210) selected disagree or strongly disagree, and the remaining 12.4% (*n* = 60) were neutral. With regard to the current limitations of AI, more students reported they had an understanding on this than those who did not; 48.3% selected strongly agree or agree to this question (7.2% *n* = 35 and 41.1% *n* = 199, respectively), whilst 30.4% selecting strongly disagree or disagree (5.2% *n* = 25 and 25.2% *n* = 122, respectively), and 21.3% (*n* = 103) selected neutral. More students reported that they did not feel comfortable with the nomenclature associated with AI (disagree and strongly disagree, 43.4% *n* = 210), than those that reported otherwise (agree and strongly agree, 30.2% *n* = 146) (Fig. 2).

**Fig. 3:**
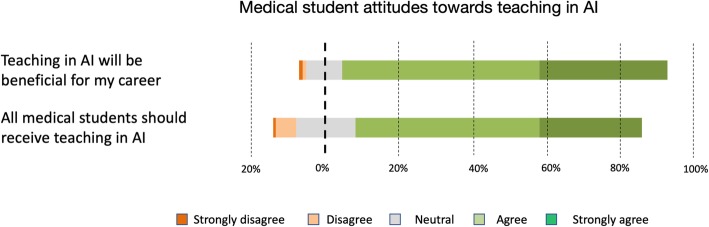
Summary of medical student attitudes towards teaching on AI

An overwhelming majority of students believed that teaching in AI would be beneficial for their careers with 88.8% students responding either strongly agree (35.1% *n* = 170) or agree (53.7% *n* = 260); 9.7% (*n* = 47) of students submitted neutral responses on this question. 1.2% of students (*n* = 6) disagreed with this statement. Along this line of questioning, a significant majority of students (78.1%) also agreed with the statement that all medical students should receive training in AI as part of their medical degree; 28.1% (*n* = 136) and 50% (*n* = 242) of students selected strongly agree and agree, respectively. Neutral responses were recorded by 16.1% (*n* = 78) of students, and only 5.6% (*n* = 27) of students responded negatively when asked if all students should receive training in AI (Fig. 3).

**Fig. 4:**
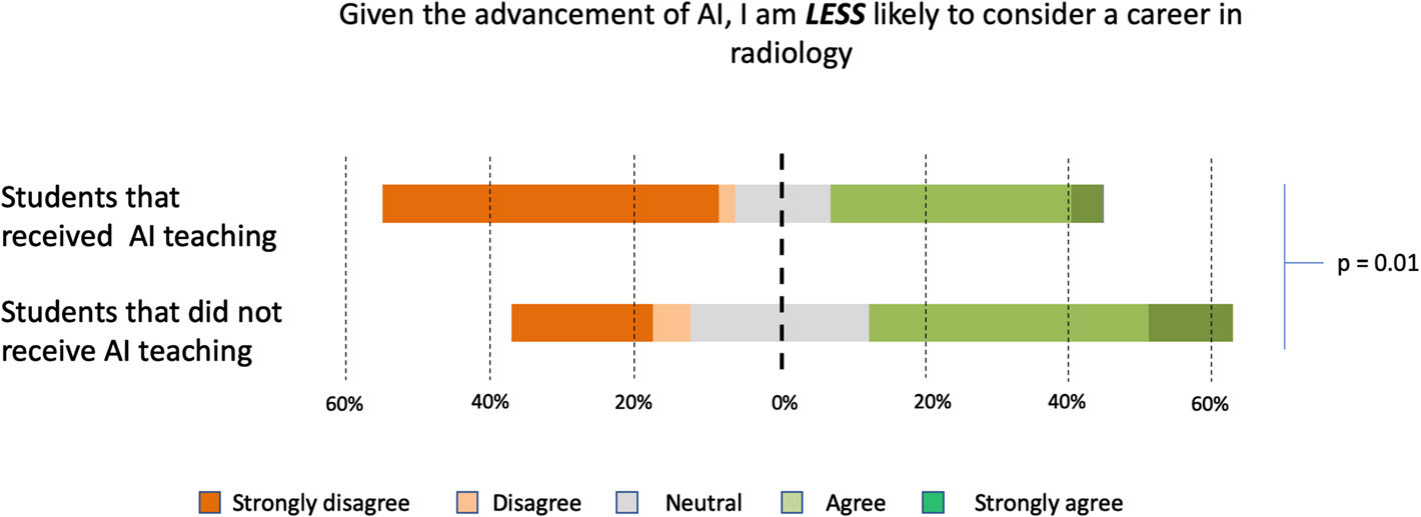
Comparison of the likelihood of considering radiology as a career between students who receive AI teaching against those that did not

Out of a total of 484 students, only 45 (9.2% of students surveyed) received some form of teaching on AI. Students from 12 out of the 19 medical schools reported teaching opportunities relating to AI, but none of these students received such teaching as a mandatory part of the curriculum. 86.7% (*n* = 39) of students found that teaching in AI was useful. Students who received no teaching were less likely to consider radiology as a possible career choice in the future compared to students who received teaching in AI (Wilcox test, *p* = 0.01) (Fig. 4).

**Fig. 5:**
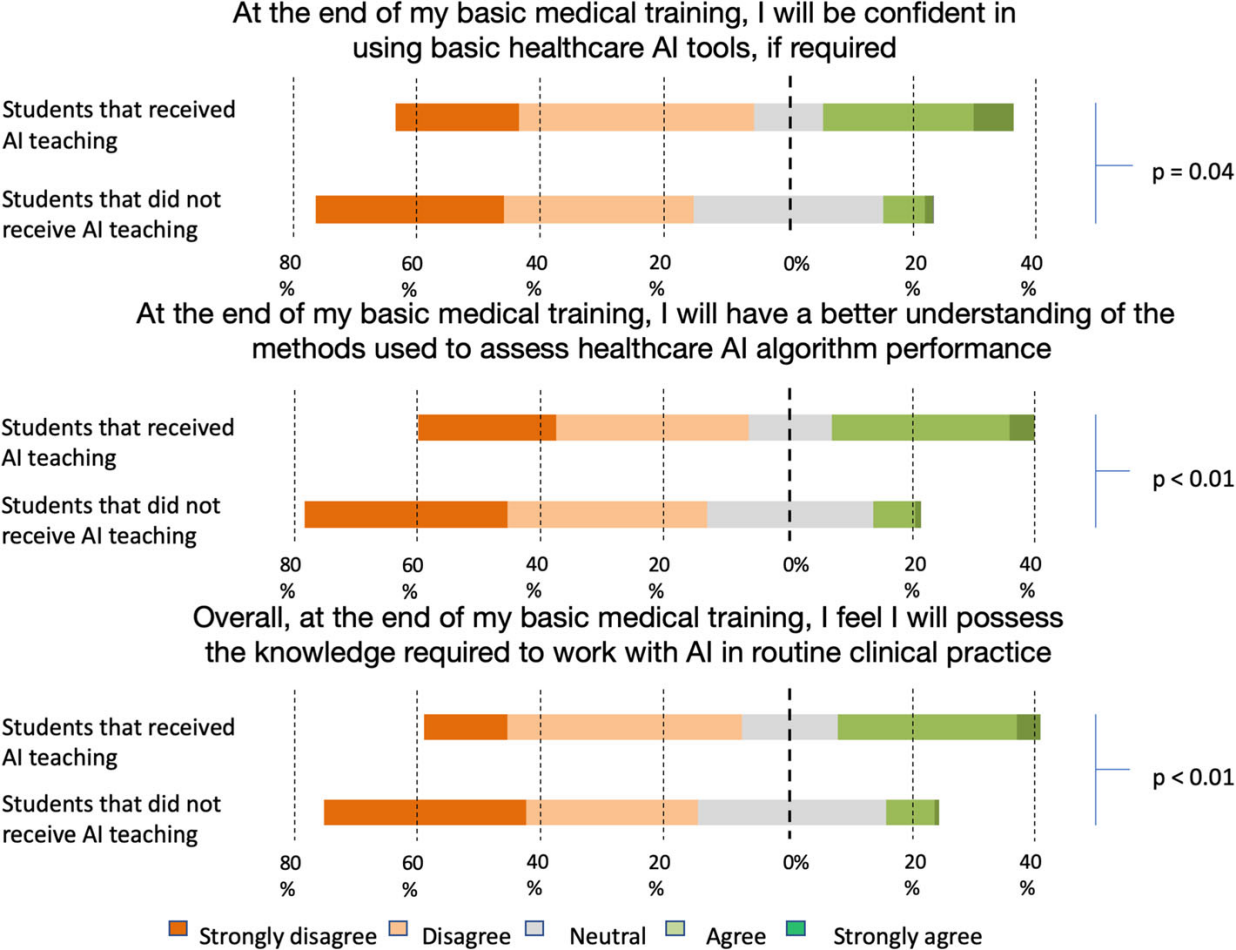
Comparison of perceived preparedness in the critical use of AI between students who receive AI teaching against those that did not

The majority of students reported they would not feel ready to work with AI by the end of their degree, only 10.4% (*n* = 50) of students agreed that they would be confident in using AI tools if required, and the same number of students agreed that they would possess a basic understanding in the methods used to assess AI performance. A slightly larger number of students felt they would possess the knowledge needed to work with AI in routine clinical practice (11.3% *n* = 55). Students who receive training in AI recorded more positive responses to these questions, rating themselves as being more confident in the use of basic AI clinical tools if required (*p* = 0.04), possess a better understanding in the assessment of AI algorithm performance (*p* = < 0.01), and overall felt more likely to have the knowledge required to work with AI in routine clinical practice (*p* = < 0.01) (Fig. 5).

## Discussion

It is now accepted that AI will likely have a profound impact in the future practice of radiology and other medical fields. This sentiment is shared by a majority of students in our cohort with 88% agreeing that AI will play an important role in healthcare. Despite convincing arguments that AI will not replace radiologists [13], almost half of the students in our study are less likely to consider a career in radiology due to the perceived success of AI. In our cohort, 48.3% of students reported that they believed certain specialties would be replaced by AI. Adverse impact on radiology recruitment secondary to AI advancement was previously demonstrated by Gong et al. in a Canadian cohort (*N* = 322) [9], where one sixth of medical students interested in radiology were discouraged from applying for a residency post. The Canadian study identified that medical students were more concerned about the displacement, rather than replacement, of radiologists which could lead to reduced workforce demands. Interestingly, a separate study from Germany by Pinto dos Santos et al (2019) (*N* = 263) [10] showed that their cohort of students did not believe that AI would replace radiologists. Although they did not examine the career intentions of the students they surveyed, it can be indirectly inferred that German students may be less likely to rule out radiology due to AI. Our results provide evidence that the advancement of AI is having a greater detrimental effect on UK medical students considering radiology as a possible career when compared to their Canadian and German counterparts. A limitation of our study is that we only sampled a fraction of all UK medical students, and not all medical schools are represented in our cohort.

It is worrying that a large number of students in our cohort are discounting radiology as a possible career given the acute shortage of radiologists in the UK [14]. The work force consequences are potentially catastrophic, especially with an ever-increasing demand for imaging investigations [14]. We believe that student perceptions on this are heavily influenced by the narrative favoured by certain ardent proponents of AI, aided by popular media. Prominent figures from the computer sciences community and venture capital have frequently billed AI as a likely ‘disruptor’ of radiologists, perpetuating the fallacy that radiologists will no longer be needed in the foreseeable future. It is only more recently that an increasingly circumspect view of AI adaptation in medical imaging has gained acceptance [13], including by some of the same thought leaders that originally predicted the rapid demise of radiologists [15]. In our opinion, the persisting misconception of AI rendering radiologists obsolete should be urgently addressed. Realistic potential use cases and limitations of this technology must be presented to medical students so that they will not feel discouraged to embrace radiology as a career. 

Pinto dos Santos et al (2019) [10] identified an overall low level of information of medical students about AI, with students stating that they acquired this from mainstream media rather than university teaching. They also highlighted that students who were more knowledgeable about AI were less afraid of working with technology. Hence, in our survey, we aimed to understand the level of AI understanding and the state of relevant education within our medical students. We considered three areas of knowledge essential in understanding the fundamentals of AI: basic understanding of the principles of AI, familiarity with associated nomenclature, and basic understanding of the current limitations of AI. Under half of the students gave responses that suggested a degree of understanding in these areas, which could explain the uncertainty of working with AI. Out of the 484 medical students surveyed, only 45 received some form of teaching in AI. None of these students received this education as part of their compulsory curriculum. It is unclear if such teaching occurred as part of their intercalated degrees, student selected modules, or self-directed learning using available online resources. It is interesting to note that the large majority of medical students in our cohort agreed that AI should be incorporated into the medical school curriculum, with a similar majority believing that an understanding on this topic would be beneficial for their careers. Our findings suggest that there is a paucity of relevant teaching amongst UK medical schools, despite the current generation of UK medical students being motivated to engage with AI and informatics teaching.

Students who did receive AI training were significantly less likely to rule out radiology as a career choice, and this may be for two reasons. Firstly, students already interested in radiology as a future career are more likely to seek information on AI to provide an informed decision on their career choice. Secondly, students who do have a better understanding of AI are likely more aware of the limitations that preclude replacement of radiologists. Furthermore, it is not unreasonable to assume that some students are encouraged by the potential opportunities that AI would present in a radiological career. Our study found that students who received teaching on the topic felt more prepared in being able to work with AI tools compared to those who did not. However, despite statistical significance between the two groups on the questions relating to post-graduation readiness on working with AI, the majority of students in the taught group still reported that they would lack both the confidence and knowledge to employ AI in a critical manner. Although most students in the taught group felt that the teaching they received is useful, our results suggest the sporadic teaching that exist within UK medical schools do not adequately prepare medical students for the impending digital revolution of healthcare. It is now accepted that AI will likely have a profound impact in the future practice of radiology and other medical fields, and it is thus inevitable that AI and other digital tools will be incorporated into clinical practice, regardless of speciality. It would be a failure if we do not seize on this opportunity to better equip our future physicians with the adequate knowledge. It is our belief that the physicians of tomorrow must possess the means to employ digital tools, including AI, in a manner that is akin to rational evidence-based medication use.

## Supplementary information

**Additional file 1.** Questionnaire on current attitudes and understanding on artificial intelligence of medical students in the United Kingdom.

## Data Availability

The survey was submitted as a supplement to the manuscript. Requests for our data can be made via the corresponding author.

## Abbreviations

AI: Artificial intelligence
CNN: Convolutional neural networks
UK: United Kingdom
