# Supplementary material for: Attitudes and perceptions of UK medical students towards artificial intelligence and radiology: a multicentre survey
Source: Insights Imaging. 2020 Feb 5;11:14. doi: 10.1186/s13244-019-0830-7 (PMC7002761; doi:10.1186/s13244-019-0830-7)
Supplement: Supplementary file 1 — Additional file 1. Questionnaire on current attitudes and understanding on artificial intelligence of medical students in the United Kingdom. [file 13244_2019_830_MOESM1_ESM.pdf]

KING'S College LONDON

NHS Guy's and St Thomas' NHS Foundation Trust

Current attitudes and understanding on artificial intelligence of medical students in the United Kingdom

Consent

Thank you for taking part!

We are a group of researchers based at Guy's and St. Thomas' NHS Foundation Trust and Kings College London who are trying to better understand your views and experiences on this topic.

This survey will approximately 3mins to complete.

Your responses will be stored securely. Only named research team members will be able to view your responses.

We aim to present the aggregate results at an academic conference and an academic journal.

By completing the survey, you consent to the above.

Which Medical School do you currently attend?

- ○ Aston Medical School
- ○ Barts and The London School of Medicine and Dentistry
- ○ University of Birmingham
- ○ Bristol Medical School
- ○ Brighton and Sussex Medical School
- ○ University of Cambridge
- ○ University of Central Lancashire
- ○ University of Exeter
- ○ Hull York Medical School
- ○ Imperial College London
- ○ Keele University
- ○ Kings College London
- ○ University of Lancaster
- ○ University of Leeds
- ○ University of Leicester
- ○ University of Liverpool
- ○ University of Manchester
- ○ University of Newcastle
- ○ University of Nottingham
- ○ University of East Anglia
- ○ University of Oxford
- ○ Plymouth University
- ○ University College London
- ○ University of Sheffield
- ○ University of Southampton
- ○ St George's, University of London
- ○ Warwick Medical School
- ○ University of Aberdeen
- ○ University of Dundee
- ○ University of Edinburgh
- ○ University of Glasgow
- ○ University of St. Andrews
- ○ University of Cardiff
- ○ Swansea University
- ○ Queens University Belfast

Please rate your agreement to the follow questions:

AI will play an important role in healthcare

- Strongly agree
- Agree
- Neither agree or disagree
- Disagree
- Strongly disagree

I am ***<u>LESS</u>*** likely to consider a career in radiology, given the advancement of AI

- Strongly agree
- Agree
- Neither agree or disagree
- Disagree
- Strongly disagree

Some specialties will be replaced by AI during my lifetime

- Strongly agree
- Agree
- Neither agree or disagree
- Disagree
- Strongly disagree

I have an understanding of the basic computational principles of AI

- Strongly agree
- Agree
- Neither agree or disagree
- Disagree
- Strongly disagree

I am comfortable with the nomenclature related to artificial intelligence

- Strongly agree
- Agree
- Neither agree or disagree
- Disagree
- Strongly disagree

I have an understanding of the limitations of artificial intelligence

- Strongly agree
- Agree
- Neither agree or disagree
- Disagree
- Strongly disagree

Teaching in artificial intelligence will be beneficial for my career

- Strongly agree
- Agree
- Neither agree or disagree
- Disagree
- Strongly disagree

All medical students should receive teaching in artificial intelligence

- Strongly agree
- Agree
- Neither agree or disagree
- Disagree
- Strongly disagree

At the end of my medical degree, I will be confident in using basic healthcare AI tools if required

- Strongly agree
- Agree
- Neither agree or disagree
- Disagree
- Strongly disagree

At the end of my medical degree, I will have a better understanding of the methods used to assess healthcare AI algorithm performance

- Strongly agree
- Agree
- Neither agree or disagree
- Disagree
- Strongly disagree

Overall, at the end of my medical degree, I feel I will possess the knowledge needed to work with AI in routine clinical practice

- Strongly agree
- Agree
- Neither agree or disagree
- Disagree
- Strongly disagree

Please answer yes or no to the following questions:

I have received teaching/training in artificial intelligence

- ○ Yes ○ No

If you have answered yes to the previous question, was this teaching/training a compulsory part of your medical degree?

- ○ Yes ○ No ○ N/A

If you have received teaching/training in artificial intelligence, please rate the usefulness of the teaching/training you have received

- ○ Extremely useful ○ Very useful ○ Somewhat useful ○ Not so useful ○ Not at all useful
